# ﻿A new species in the *Cyrtodactylusoldhami* group (Squamata, Gekkonidae) from Kanchanaburi Province, western Thailand

**DOI:** 10.3897/zookeys.1103.84672

**Published:** 2022-06-02

**Authors:** Siriporn Yodthong, Attapol Rujirawan, Bryan L. Stuart, L. Lee Grismer, Akrachai Aksornneam, Korkhwan Termprayoon, Natee Ampai, Anchalee Aowphol

**Affiliations:** 1 Department of Zoology, Faculty of Science, Kasetsart University, Bangkok 10900, Thailand Kasetsart University Bangkok Thailand; 2 North Carolina Museum of Natural Sciences, 11 West Jones Street, Raleigh, North Carolina 27601, USA North Carolina Museum of Natural Sciences Raleigh United States of America; 3 Herpetology Laboratory, Department of Biology, La Sierra University, 4500 Riverwalk Parkway, Riverside, California 92505, USA La Sierra University Riverside United States of America; 4 Department of Biology, Faculty of Science, Srinakharinwirot University, Bangkok 10110, Thailand Srinakharinwirot University Bangkok Thailand

**Keywords:** *Cyrtodactylusmonilatus* sp. nov., *Cyrtodactyluszebraicus*, integrative taxonomy, mitochondrial DNA, morphology, phylogeny, Southeast Asia

## Abstract

*Cyrtodactylusmonilatus***sp. nov.** is described from Si Sawat District, Kanchanaburi Province, in western Thailand. The new species superficially resembles *C.zebraicus* Taylor, 1962 from southern Thailand. However, differences between the new species from *C.zebraicus* and other congeners were supported by an integrative taxonomic analysis of molecular and morphological data. Phylogenetic analyses based on the mitochondrial NADH dehydrogenase subunit 2 (ND2) gene showed that the new species is a member of the *C.oldhami* group and closely related to *Cyrtodactylus* sp. MT468911 from Thong Pha Phum National Park, Thong Pha Phum District, Kanchanaburi Province. Uncorrected pairwise genetic divergences (*p*-distances) between the new species and its congeners, including *C.zebraicus*, ranged from 7.7–17.7%. *Cyrtodactylusmonilatus***sp. nov.** can also be distinguished from all members of the *C.oldhami* group by having a unique combination of morphological characters, including a snout to vent length of 53.7–63.3 mm in adult males and 58.6–75.8 mm in adult females; 22–34 paravertebral tubercles; 34–42 ventral scales; 30–39 enlarged contiguous femoroprecloacal scales; femoral pores and precloacal pores absent in both sexes; four or five rows of postprecloacal scales; enlarged median subcaudal scales absent; weak ventrolateral folds present; 4–7 rows of paired, paravertebral, dark-brown blotches edged in yellow or yellowish white; and two rows of small, diffuse, yellow or yellowish white spots on flanks. The new species occurs in a narrow range of forest at mid to low elevations associated with karst landscapes in the Tenasserim mountain range.

## ﻿Introduction

The Bent-toed Gecko genus *Cyrtodactylus* Gray, 1827 is the third largest vertebrate genus in the world and one of the most species-rich radiations of gekkonid lizards (Grismer et al. 2021b). The genus is widely distributed from South and Southeast Asia into northern Australia and Melanesia (Grismer et al. 2021b; Uetz et al. 2022), where it occupies a broad variety of habitats associated with karst landscapes and forested areas (Grismer et al. 2021a). The genus *Cyrtodactylus* is monophyletic and currently contains 330 recognized species (Uetz et al. 2022) within 32 monophyletic species groups that have been delimited based on molecular data (Grismer et al. 2022). Most of the known species diversity is in mainland Southeast Asia (Wood et al. 2012; Grismer et al. 2018b, 2021b; Uetz et al. 2022), including Thailand, which is home to 39 species or nearly 9% of the described diversity (e.g., Chomdej et al. 2020; Grismer et al. 2020; Termprayoon et al. 2021; Uetz et al. 2022). Although the number of recognized species in the genus has rapidly increased in recent years, the true species diversity of the genus is still underestimated, and many known molecular lineages await formal description as species (Brennan et al. 2017; Chomdej et al. 2021; Grismer et al. 2021b).

The *Cyrtodactylusoldhami* group is restricted to a narrow geographic range on the Thai-Malay Peninsula and Myanmar northward into Kanchanaburi Province in western Thailand (Panitvong et al. 2014; Pauwels et al. 2016; Connette et al. 2017; Grismer et al. 2018b, 2021b). The *oldhami* group is one of the most taxonomically diverse species groups of *Cyrtodactylus* in Thailand. The group is monophyletic and contains at least seven nominal species (Grismer et al. 2021b), of which five occur in Thailand, i.e., *C.oldhami* (Theobald, 1876), *C.saiyok* Panitvong, Sumontha, Tunprasert & Pauwels, 2014, *C.sanook* Pauwels, Sumontha, Latinne & Grismer, 2013, *C.thirakhupti* Pauwels, Bauer, Sumontha & Chanhome, 2004, and *C.zebraicus* Taylor, 1962. *Cyrtodactylusphetchaburiensis* Pauwels, Sumontha & Bauer, 2016 from southern Thailand may also belong to the *oldhami* group, based on morphological characters (Pauwels et al. 2016), but this hypothesis remains untested by molecular data. Two additional species in the group are known from Myanmar, i.e., *C.lenya* Mulcahy, Thura & Zug, 2017, and *C.payarhtanensis* Mulcahy, Thura & Zug, 2017. The members of the *oldhami* group are morphologically variable, especially in color pattern, but the group is morphologically diagnosable from the other species groups (see Grismer et al. 2018b).

*Cyrtodactyluspeguensiszebraicus* Taylor, 1962 was originally described from Ron Phibun (“Ronpibon”) District, Nakhon Si Thammarat Province in southern Thailand. The taxonomic status of this species was long uncertain, and often confused with *C.peguensis* (Boulenger, 1893). Grismer et al. (2018a) redescribed *C.peguensis* based on new collections from its type (and only known) locality at Bago Region, Taikkyi Township, Yangon (north) District, Myanmar. In addition, they also studied specimens of *C.peguensiszebraicus* from southern Thailand (Nakhon Si Thammarat, Surat Thani, and Trang provinces) and compared them to other species in the *C.peguensis* group. Their phylogenetic results revealed that *C.zebraicus* is not closely related to *C.peguensis*, but rather belongs within the *C.oldhami* group. As such, Grismer et al. (2018a) elevated *C.peguensiszebraicus* to full species status (as *C.zebraicus*) and removed it from the synonymy of *C.peguensis*.

During our fieldwork in 2019 and 2021, we collected *Cyrtodactylus* specimens of the *C.oldhami* group from three localities in Si Sawat District, Kanchanaburi Province, western Thailand. These specimens closely resembled *C.zebraicus* in body size, color pattern and habitat usage. The taxonomic status of the Si Sawat specimens was investigated using mitochondrial DNA and morphological data. The datasets corroborated differences in the Si Sawat specimens from *C.zebraicus* and other species of the *C.oldhami* group. Herein, we describe this population as a new species.

## ﻿Materials and methods

### ﻿Sampling

**Figure 1.**
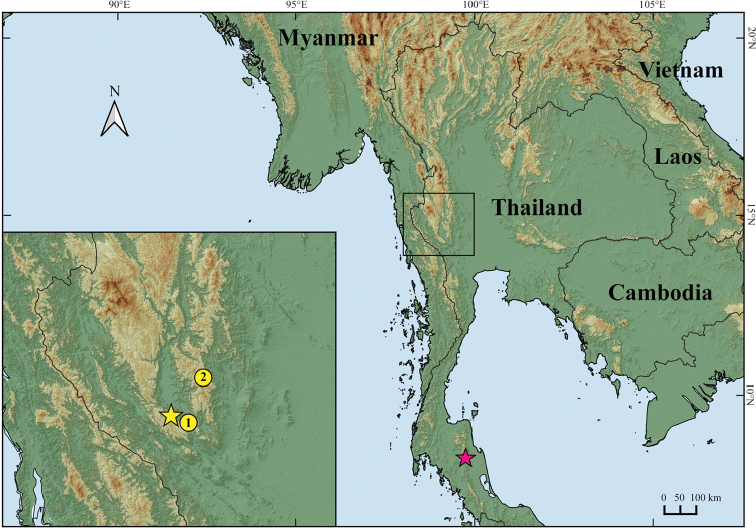
Map illustrating the type locality at Tham Phrathat Protection Unit (yellow star) and paratype localities (yellow cycle) at Erawan Waterfall (1) and at Tham Than Lot Noi-Tham Than Lot Yai Nature Trail (2), Si Sawat District, Kanchanaburi Province, Thailand of *Cyrtodactylusmonilatus* sp. nov., and the type locality (pink star) at Ron Phibun (= “Ronpibon”) District, Nakhon Si Thammarat Province, Thailand of *C.zebraicus* (“*C.peguensiszebraicus*”).

A total of 22 specimens (eleven adult males, nine adult females, and two juveniles) of the Si Sawat *Cyrtodactylus* were collected by hand during fieldwork in April and November 2019, and November 2021 from Si Sawat District, Kanchanaburi Province, western Thailand (Fig. 1). Geographical coordinates and elevation were recorded with a Garmin GPSMAP 64s. Ambient air temperature and relative humidity were collected using a Kestrel 4000 Weather Meter. Photographs were taken to document the color pattern of specimens in life prior to preservation. The specimens were humanely euthanized using cardiac injection of tricaine methanesulfonate (MS-222) solution (Simmons 2015). Liver tissue was collected from each individual, preserved in 95% ethyl alcohol, and stored at -20 °C for genetic analysis. Voucher specimens were then initially fixed in 10% buffered formalin and later transferred to 70% ethyl alcohol for long-term preservation. All specimens were deposited in the herpetological collection of the Zoological Museum, Kasetsart University, Bangkok, Thailand (**ZMKU**). The holotype of *C.zebraicus* was examined in the holdings of the Field Museum of Natural History, Chicago (**FMNH**).

### ﻿Molecular analyses

Total genomic DNA were extracted from preserved liver tissue of nine individuals of the Si Sawat species (Table 1) using the NucleoSpin Tissue DNA Extraction Kit (Macherey-Nagel Inc., Düren, Germany). A 1,355–1,394 base pair (bp) fragment of mitochondrial (mt) DNA that encodes the complete NADH dehydrogenase subunit 2 (ND2) gene and partial flanking tRNA genes was amplified by the polymerase chain reaction (PCR) under the following conditions: initial denaturation at 95 °C for 2 min, followed by 33 cycles of a second denaturation at 95 °C for 35 s, annealing at 54 °C for 35 s, extension at 72 °C for 35 s, and final extension at 72 °C for 10 min using the primer pair L4437b (AAGCTTTCGGGCCCATRCC; Macey et al. 1997) and H5934 (AGRGTGCCAATGTCTTTGTGRTT; Macey et al. 1997). PCR products were purified using the QIAquick PCR Purification Kit (Qiagen Ltd., Hilden, Germany) and sequenced in both directions on an ABI 3730XL sequencers by Sangon Biotech Inc. (Shanghai, China) using BigDye version 3 chemistry and the amplifying primers. DNA sequences were edited and aligned using Geneious R11 (Biomatter, Ltd., Auckland, New Zealand). The protein-coding region of the ND2 sequence was aligned and translated to amino acids to verify that the desired protein-coding genes were correctly sequenced and edited. All novel sequences were deposited in GenBank under the accession numbers ON231266–ON231274 (Table 1).

**Table 1.** Specimens used in this study, including localities, museum numbers and GenBank accession numbers of the mitochondrial NADH dehydrogenase subunit 2 gene and flanking tRNA regions.

Species	Locality	Voucher No.	Accession No.	Reference
**Ingroup**				
*Cyrtodactylusamphipetraeus*	Tha Ra Rak Waterfall, Mae Sot Dist., Tak Prov., Thailand	ZMMU R 16626	MT550630	Chomdej et al. (2020)
*Cyrtodactylusbrevipalmatus*	Khao Ramrome, Ron Phibun Dist., Nakhon Si Thammarat Prov., Thailand	AUP-00573	MT468899	Chomdej et al. (2021)
*Cyrtodactylusdammathetensis*	Dammathet Cave, 19.8 km east of Mawlamyine, Mawlamyine Dist., Mon State, Myanmar	LSUHC 12864	MF872278	Grismer et al. (2018b)
*Cyrtodactylusdoisuthep*	Doi Suthep, Mueang Dist., Chiang Mai Prov., Thailand	AUP-00777	MT497801	Chomdej et al. (2021)
*Cyrtodactylusdumnuii*	Chiang Dao, Chiang Mai Prov., Thailand	AUP-00768	MW713972	Grismer et al. (2021b)
*Cyrtodactyluserythrops*	Coral Cave, Pang Mapha Dist., Mae Hong Son Prov., Thailand	AUP-00771	MT497806	Chomdej et al. (2021)
*Cyrtodactylusinterdigitalis*	Nakai Dist., Khammouan Prov., Laos	FMNH 255454	JQ889181	Johnson et al. (2012)
*Cyrtodactylusinthanon*	Tiger Head mountain, Doi Inthanon National Park, Chom Thong Dist., Chiang Mai Prov., Thailand	AUP-00156	MT497800	Chomdej et al. (2021)
*Cyrtodactyluslenya*	The proposed Lenya National Park Extension, Tanintharyi Region, Myanmar	USNM 587789	KY041652	Connette et al. (2017)
*Cyrtodactyluslenya*	The proposed Lenya National Park Extension, Tanintharyi Region, Myanmar	USNM 587788	KY041653	Connette et al. (2017)
*Cyrtodactyluslenya*	The proposed Lenya National Park Extension, Tanintharyi Region, Myanmar	CAS 260233	KY041655	Connette et al. (2017)
*Cyrtodactyluslinnoensis*	Linno Cave region 5 km south-west of Hpa-an, Hpa-an Dist., Kayin State, Myanmar	LSUHC 12825	MF872295	Grismer et al. (2018b)
*Cyrtodactyluslinnwayensis*	12.7 km north-east of Ywangan, Linn-Way Village, Yum Twing Gyi Cave, Taunggyi Dist., Shan State, Myanmar,	BYU 52214	MF872280	Grismer et al. (2018b)
*Cyrtodactylusmaelanoi*	Tha Pha Pum Subdist., Mae La Noi Dist., Mae Hong Son Prov., Thailand	ZMKU R 00858	MT823267	Grismer et al. (2020)
***Cyrtodactylusmonilatus* sp. nov.**	Chaloem Rattanakosin National Park, Khao Chot Subdist., Si Sawat Dist., Kanchanaburi Prov., Thailand	ZMKU R 00923	–	This study
***Cyrtodactylusmonilatus* sp. nov.**	Chaloem Rattanakosin National Park, Khao Chot Subdist., Si Sawat Dist., Kanchanaburi Prov., Thailand	ZMKU R 00924	–	This study
***Cyrtodactylusmonilatus* sp. nov.**	Chaloem Rattanakosin National Park, Khao Chot Subdist., Si Sawat Dist., Kanchanaburi Prov., Thailand	ZMKU R 00925	–	This study
***Cyrtodactylusmonilatus* sp. nov.**	Chaloem Rattanakosin National Park, Khao Chot Subdist., Si Sawat Dist., Kanchanaburi Prov., Thailand	ZMKU R 00926	ON231266	This study
***Cyrtodactylusmonilatus* sp. nov.**	Erawan National Park, Tha Kradan Subdist., Si Sawat Dist., Kanchanaburi Prov., Thailand	ZMKU R 00927	ON231267	This study
***Cyrtodactylusmonilatus* sp. nov.**	Erawan National Park, Tha Kradan Subdist., Si Sawat Dist., Kanchanaburi Prov., Thailand	ZMKU R 00928	–	This study
***Cyrtodactylusmonilatus* sp. nov.**	Erawan National Park, Tha Kradan Subdist., Si Sawat Dist., Kanchanaburi Prov., Thailand	ZMKU R 00929	–	This study
***Cyrtodactylusmonilatus* sp. nov.**	Erawan National Park, Tha Kradan Subdist., Si Sawat Dist., Kanchanaburi Prov., Thailand	ZMKU R 00930	–	This study
***Cyrtodactylusmonilatus* sp. nov.**	Erawan National Park, Tha Kradan Subdist., Si Sawat Dist., Kanchanaburi Prov., Thailand	ZMKU R 00931	–	This study
***Cyrtodactylusmonilatus* sp. nov.**	Erawan National Park, Tha Kradan Subdist., Si Sawat Dist., Kanchanaburi Prov., Thailand	ZMKU R 00932	–	This study
***Cyrtodactylusmonilatus* sp. nov.**	Erawan National Park, Tha Kradan Subdist., Si Sawat Dist., Kanchanaburi Prov., Thailand	ZMKU R 00933	–	This study
***Cyrtodactylusmonilatus* sp. nov.**	Erawan National Park, Tha Kradan Subdist., Si Sawat Dist., Kanchanaburi Prov., Thailand	ZMKU R 00934	–	This study
***Cyrtodactylusmonilatus* sp. nov.**	Erawan National Park, Tha Kradan Subdist., Si Sawat Dist., Kanchanaburi Prov., Thailand	ZMKU R 00935	–	This study
***Cyrtodactylusmonilatus* sp. nov.**	Erawan National Park, Tha Kradan Subdist., Si Sawat Dist., Kanchanaburi Prov., Thailand	ZMKU R 00936	ON231268	This study
***Cyrtodactylusmonilatus* sp. nov.**	Erawan National Park, Tha Kradan Subdist., Si Sawat Dist., Kanchanaburi Prov., Thailand	ZMKU R 00937	–	This study
***Cyrtodactylusmonilatus* sp. nov.**	Erawan National Park, Tha Kradan Subdist., Si Sawat Dist., Kanchanaburi Prov., Thailand	ZMKU R 00938	–	This study
***Cyrtodactylusmonilatus* sp. nov.**	Erawan National Park, Tha Kradan Subdist., Si Sawat Dist., Kanchanaburi Prov., Thailand	ZMKU R 00939	ON231269	This study
***Cyrtodactylusmonilatus* sp. nov.**	Erawan National Park, Tha Kradan Subdist., Si Sawat Dist., Kanchanaburi Prov., Thailand	ZMKU R 00940	ON231270	This study
***Cyrtodactylusmonilatus* sp. nov.**	Erawan National Park, Tha Kradan Subdist., Si Sawat Dist., Kanchanaburi Prov., Thailand	ZMKU R 00941	ON231271	This study
***Cyrtodactylusmonilatus* sp. nov.**	Erawan National Park, Tha Kradan Subdist., Si Sawat Dist., Kanchanaburi Prov., Thailand	ZMKU R 00942	ON23172	This study
***Cyrtodactylusmonilatus* sp. nov.**	Erawan National Park, Tha Kradan Subdist., Si Sawat Dist., Kanchanaburi Prov., Thailand	ZMKU R 00943	ON231273	This study
***Cyrtodactylusmonilatus* sp. nov.**	Erawan National Park, Tha Kradan Subdist., Si Sawat Dist., Kanchanaburi Prov., Thailand	ZMKU R 00944	ON231274	This study
Cyrtodactyluscf.oldhami	Suan Phueng Distc., Ratchburi Prov., Thailand	HLM 0307	MW713967	Grismer et al. (2021b)
*Cyrtodactylusoldhami*	Kraburi Dist., Phang-nga Prov., Thailand	MS 460	MF872301	Grismer et al. (2018b)
*Cyrtodactylusoldhami*	Muang Dist., Ranong Prov., Thailand	MS 585	MF872302	Grismer et al. (2018b)
*Cyrtodactylusoldhami*	Chumpon Prov., Thailand	LSUHC 9486	MH940241	Murdoch et al. (2019)
*Cyrtodactyluspayarhtanensis*	in the proposed Lenya National Park, Tanintharyi Region, Myanmar	USNM 587409	KY041656	Connette et al. (2017)
*Cyrtodactyluspayarhtanensis*	in the proposed Lenya National Park, Tanintharyi Region, Myanmar	USNM 587792	KY041657	Connette et al. (2017)
*Cyrtodactyluspayarhtanensis*	in the proposed Lenya National Park, Tanintharyi Region, Myanmar	USNM 587791	KY041658	Connette et al. (2017)
*Cyrtodactyluspharbaungensis*	Pharpoun Cave, 38.4 km south-east of Mawlamyine, Mawlamyine Dist., Mon State, Myanmar	BYU 52215	MF872303	Grismer et al. (2018b)
*Cyrtodactylussadanensis*	Sadan Cave, 17 km south-east of Hpa-an, Hpa-an Dist., Kayin State, Myanmar	LSUHC 12853	MF872324	Grismer et al. (2018b)
*Cyrtodactylussadansinensis*	Sadan Sin Cave 10.5 km north-west of Mawlamyine, Mawlamyine Dist., Mon State, Myanmar	BYU 52220	MF872325	Grismer et al. (2018b)
*Cyrtodactylussaiyok*	Sai Yok National Park, Kanchanaburi Prov., Thailand	MS 484	MF872308	Grismer et al. (2018b)
*Cyrtodactylussaiyok*	Sai Yok National Park, Kanchanaburi Prov., Thailand	MS 480	MF872309	Grismer et al. (2018b)
*Cyrtodactylussaiyok*	Sai Yok Dist., Kanchanaburi Prov., Thailand	AUP-00773	MT497805	Chomdej et al. (2021)
*Cyrtodactylussanook*	Tham Sanook, Muang Dist., Chumphon Prov., Thailand	AUP-00570	MT468898	Chomdej et al. (2021)
*Cyrtodactylussanpelensis*	Sanpel Cave, 21.3 km south-east of Mawlamyine, Mawlamyine Dist., Mon State, Myanmar	LSUHC 12886	MF872343	Grismer et al. (2018b)
*Cyrtodactylusshwetaungorum*	5.3 km north of Pyinyaung Village at the Apache Cement factory mining site, Mandalay Region, Myanmar	BYU 52227	MF872348	Grismer et al. (2018b)
*Cyrtodactylus* sp.	Moe Cham Pae Dist., Mae Hong Son Prov., Thailand	HLM 0357	MW713961	Grismer et al. (2021b)
*Cyrtodactylus* sp.	Krabi, Trang Prov., Thailand	HLM 0358	MW713969	Grismer et al. (2021b)
*Cyrtodactylus* sp. MT468910	Thong Pha Phum National Park, Thong Pha Phum Dist., Kanchanaburi Prov., Thailand	AUP-01718	MT468910	Chomdej et al. (2021)
*Cyrtodactylu*s sp. MT468911	Near Vajiralongkorn dam, Thong Pha Phum National Park, Thong Pha Phum Dist., Kanchanaburi Prov., Thailand	AUP-01722	MT468911	Chomdej et al. (2021)
*Cyrtodactylusthirakhupti*	Tham Khao Sonk hill, Surat Thani Prov., Thailand	ZMKU R 00732/LSUHC 12467	MF872357	Grismer et al. (2018b)
*Cyrtodactylusthirakhupti*	Tham Khao Sonk hill, Surat Thani Prov., Thailand	ZMKU R 00733/ LSUHC 12468	MF872358	Grismer et al. (2018b)
*Cyrtodactylustigroides*	Ban Tha Sao, Sai-Yok Dist., Kanchanaburi Prov., Thailand	IRSNB2380	JX440562	Wood et al. (2012)
*Cyrtodactylustigroides*	Wang Krachae Subdist., Sai Yok Dist., Kanchanaburi Prov., Thailand	AUP-00776	MT497804	Chomdej et al. (2021)
*Cyrtodactylusyathepyanensis*	Yathe Pyan Cave, 9 km south-west of Hpa-an, Hpa-an Dist., Kayin State, Myanmar	LSUHC 12823	MF872367	Grismer et al. (2018b)
*Cyrtodactyluszebraicus*	Mueang Krabi, Krabi, Thailand	HLM 0344	MW713971	Grismer et al. (2021b)
*Cyrtodactyluszebraicus*	Khao Luang National Park, Thailand	CUMZR 2005.07.30.54	GU550727	Siler et al. (2010)
*Cyrtodactyluszebraicus*	Ron Phibun, Nakhon Si Thammarat, Thailand	FMNH 178286	–	This study
**Outgroup**				
*Dixoniussiamensis*	Thong Pha Phum National Park, Thong Pha Phum Dist., Kanchanaburi Prov., Thailand	AUP-01724	MT468896	Chomdej et al. (2021)
*Gekkogecko*	Shwesettaw wildlife sanctuary, Mimbu Township, Magway Div., Myanmar	CAS 213628	JN019053	RÖsler et al. (2011)
*Gekkokaengkrachanense*	Thong Pha Phum National Park, Thong Pha Phum Dist., Kanchanaburi Prov., Thailand	AUP-01710	MT468895	Chomdej et al. (2021)
*Hemidactylusfrenatus*	Rathegala, Sri Lanka	AMB 7420	EU268359	Bauer et al. (2008)

All available ND2 sequences of related species in the genus *Cyrtodactylus* from Myanmar-Thai populations and the outgroups *Dixoniussiamensis* (Boulenger, 1899), *Gekkogecko* (Linnaeus, 1758), *G.kaengkrachanense* (Sumontha, Pauwels, Kunya, Limlikhitaksorn, Ruksue, Taokratok, Ansermet & Chanhome, 2012), and *Hemidactylusfrenatus* Duméril & Bibron, 1836 were downloaded from GenBank following Grismer et al. (2018b, 2021b) and Chomdej et al. (2021) (Table 1). The downloaded sequences were aligned to the newly-generated sequences of the new species using the MUSCLE plug-in as implemented in Geneious R11. The alignment was edited by eye and trimmed with the gaps partially deleted to ensure that did not disrupt the coding region. Phylogenetic relationships were constructed using Maximum Likelihood (ML) and Bayesian Inference (BI) analysis. The dataset was partitioned by codon position and a separate partition for the tRNAs. The best partitioning scheme and models of evolution were selected using ModelFinder function in IQ-TREE (Kalyaanamoorthy et al. 2017) with the Bayesian Information Criterion (BIC). The best-fit partitioning scheme and models of evolution are listed in Table 2. The ML analysis was performed using the IQ-TREE webserver (Trifinopoulos et al. 2016), with 1,000 bootstrap pseudoreplicates using the ultrafast bootstrap (UFB) approximation algorithm (Hoang et al. 2017). Nodes with UFB ≥ 95 were considered to be strongly supported (Minh et al. 2013).

**Table 2.** Models of molecular evolution selected for the maximum likelihood and Bayesian analyses.

Gene	Model selected	Model applied for ML	Model applied for BI
**ND2**			
1^st^ position	TVM+F+I+G4	TVM+F+I+G4	GTR+I+Γ
2^nd^ position	TPM3u+F+I+G4	TPM3u+F+I+G4	GTR+I+Γ
3^rd^ position	TIM3+F+G4	TIM3+F+G4	GTR+I+Γ
**tRNAs**	TIMe+G4	TIMe+G4	GTR+I+Γ

The BI analysis was performed on CIPRES Science Gateway (Miller et al. 2010) using MrBayes v3.2.6 on XSEDE (Ronquist et al. 2012) with the partitioning scheme and models of evolution most closely approximating those calculated in IQ-TREE for the ML analysis. Two simultaneous runs each with four chains were performed using Markov chain Monte Carlo (MCMC). MCMC chains were run for 20 million generations using the default priors, chain temperature set to 0.1, trees sampled every 1,000 generations, and the first 25% of trees discarded as burn-in. The convergence of the two simultaneous runs, and stationary states of each parameter, were assessed based on the standard deviation of split frequencies (< 0.01) and the effective sample sizes (ESS) scores were above 200 in Tracer v1.7.1 (Rambaut et al. 2018). A 50% majority-rule consensus of the sampled trees was constructed to calculate the posterior probabilities (PP) of the tree nodes. Nodes with PP ≥ 0.95 were considered to be strongly supported (Huelsenbeck and Ronquist 2001; Wilcox et al. 2002). Uncorrected pairwise sequence divergences (*p*-distances) were calculated in MEGA 11 (Tamura et al. 2021) using the pairwise deletion option.

### ﻿Morphological analyses

Mensural, meristic, and qualitative characters were taken using a Nikon SMZ 745 Zoom Stereomicroscope. Measurements were taken on the left side of the body when possible, with digital calipers (Mitutoyo CD-6” ASX Digimatic Caliper, Japan) to the nearest 0.1 mm. Characters and abbreviations were modified from Grismer et al. (2018a, 2018b). Morphological measurements were as follows:

**SVL** Snout to vent length, taken from the tip of snout to the vent;

**HL** Head length, the distance from the posterior margin of the retroarticular process of the lower jaw to the tip of the snout;

**HW** Head width, measured at the angle of the jaws;

**HD** Head depth, the maximum height of head measured from the occiput to the mandibles;

**ED** Eye diameter, the greatest horizontal diameter of the eyeball;

**EE** Eye to ear distance, measured from the anterior edge of the ear opening to the posterior edge of the eyeball;

**ES** Eye to snout distance, measured from anterior most margin of the eyeball to the tip of snout;

**EN** Eye to nostril distance, measured from the anterior margin of the eyeball to the posterior margin of the external nares;

**IO** Interorbital distance, measured between the anterior edges of the orbit;

**EL** Ear diameter, the greatest vertical distance of the ear opening;

**IN** Internarial distance, measured between the nares across the rostrum;

**FL** Forearm length, taken on the dorsal surface from the posterior margin of the elbow while flexed 90° to the inflection of the flexed wrist;

**TBL** Tibia length, taken on the ventral surface from the posterior surface of the knee while flexed 90° to the base of the heel;

**AG** Axilla to groin length, taken from the posterior margin of the forelimb at its insertion point on the body to the anterior margin of the hind limb at its insertion point on the body;

**TL** Tail length, taken from the vent to the tip of the tail, original and regenerated;

**TW** Tail width, taken at the base of the tail immediately posterior to the postcloacal swelling.

Meristic characters were taken on both right and left (R/L) sides when possible. The presence, absence, and/or numbers of the characters were recorded as follows:

**SL** The numbers of supralabial scales, counted from the largest scale immediately below the posterior margin of the eyeball to the rostral scales;

**SL-mideye** The numbers of supralabial scales, counted from the largest scale immediately below the middle of the eyeball to the rostral scales;

**IL** The numbers of infralabial scales, counted from the largest scale immediately below the posterior margin of the eyeball to the mental scales;

**IL-mideye** The numbers of infralabial scales, counted from the largest scale immediately below the middle of the eyeball to the mental scales;

**PVT** The number of paravertebral tubercles between limb insertions, counted in a straight line immediately left of the vertebral column;

**LRT** The number of longitudinal rows of dorsal tubercles, counted transversely across the center of the dorsum from one ventrolateral fold to the other;

**VS** The number of longitudinal rows of ventral scales, counted transversely across the center of the abdomen from one ventrolateral fold to the other;

**4FLE** The number of expanded subdigital lamellae proximal to the digital inflection on the fourth finger, counted from the base of the first phalanx where it contacts the body of the hand to the largest scale on the digital inflection;

**4FLU** The number of small, unmodified subdigital lamellae distal to the digital inflection on the fourth finger, counted from the digital inflection to the claw;

**4FL** The total number of subdigital lamellae beneath the fourth finger;

**4TLE** The number of expanded subdigital lamellae proximal to the digital inflection on the fourth toe, counted from the base of the first phalanx where it contacts the body of the foot to the largest scale on the digital inflection;

**4TLU** The number of small, unmodified subdigital lamellae distal to the digital inflection on the fourth toe, counted from the digital inflection to the claw;

**4TL** The total number of subdigital lamellae beneath the fourth toe;

**FPS** The number continuous femoroprecloacal scales in males and females;

**PP** Presence or absence precloacal pores in males and females;

**PPS** The number of rows of post-precloacal scales on the midline between the enlarged precloacal scales and the vent;

**PPT** The number of postcloacal tubercles;

**BB** The number of body bands between the nuchal loop (dark band running from eye to eye) and the hind limb insertions not including the nape or postsacral bands;

**LCB** The number of light caudal bands on an original tail;

**DCB** The number of dark caudal bands on an original tail.

Non-meristic morphological characters examined were the degree of body tuberculation, weak tuberculation refers to low and weakly keeled dorsal body tubercles whereas prominent tuberculation refers to raised and prominently keeled dorsal body tubercles; body tubercles extending past the base of the tail or not; enlarged femoral scales and precloacal scales contiguous or separated by a diastema at the base of the femora; a precloacal depression or groove present or absent; transversely expanded, median subcaudal scales present or absent; and the relative length to width ratio of the transversely expanded, median subcaudal scales. Color pattern characters evaluated were the nuchal loop being continuous from eye to eye, or separated medially into paravertebral blotches; the dorsal body bands bearing paired, paravertebral elements or fused medially; dark dorsal body bands edged with light-colored tubercles or not; dark markings present or absent in the dorsal interspace; ventrolateral body folds weak or prominent; top of head bearing combinations of dark diffuse mottling or dark distinct blotches overlain with a light-colored reticulating network or not; light-colored caudal bands encircling tail or not; and regenerated tail bearing a pattern of distinct, dark spots or not.

Morphological comparisons were based on examination of the holotype of *C.zebraicus* (FMNH 178286) as well as data taken from the original and expanded descriptions of species in the literature (Theobald 1876; Taylor 1962; Pauwels et al. 2004; Pauwels et al. 2013; Panitvong et al. 2014; Connette et al. 2017; Grismer et al. 2018a).

## ﻿Results

### ﻿Molecular analyses

**Figure 2.**
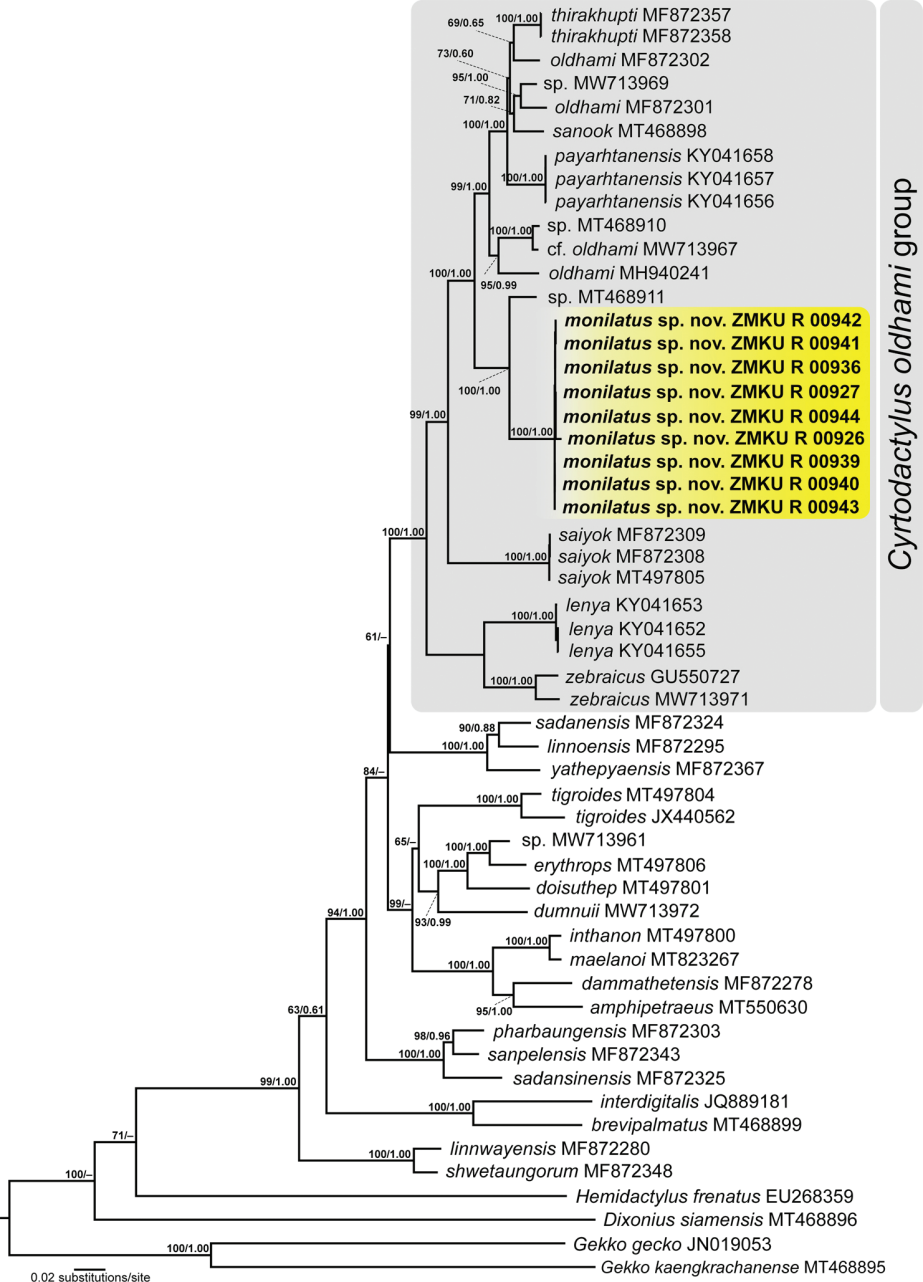
The best maximum likelihood tree showing the relationships of the *Cyrtodactylusoldhami* group and other related species distributed in Southeast Asia based on 1,444 bp of the ND2 gene and flanking tRNAs. Support values on branches are ultrafast bootstrap (UFB) followed by posterior probabilities (PP) resulting from a separate partitioned Bayesian analysis.

The total aligned dataset contained 1,444 mtDNA characters with gaps from 50 individuals of *Cyrtodactylus* species and four individuals of the outgroup species. The standard deviation of split frequencies among the two BI runs was 0.001410, and the ESS of all parameters were ≥ 10,325.8, indicating that the two runs had been sufficiently sampled and converged. The maximum likelihood value of the best ML tree was lnL = -19,837.548. The 50% majority rule consensus tree from BI analysis and the best ML tree had identical ingroup topologies, and so the ML topology was used herein (Fig. 2).

In both analyses, the Si Sawat species represented a deeply divergent mtDNA lineage and a strongly supported monophyletic group (1.0 PP, 100 UFB; Fig. 2) nested within the *C.oldhami* group containing *C.lenya*, *C.oldhami*, *C.payarhtanensis*, *C.sanook*, *C.saiyok*, *Cyrtodactylus* sp. MW713969, *Cyrtodactylus* sp. MT468910, *Cyrtodactylus* sp. MT468911, *C.thirakhupti*, and *C.zebraicus*. The Si Sawat species was strongly supported as the sister taxon to *Cyrtodactylus* sp. MT468911 from Thong Pha Phum National Park, Thong Pha Phum District, Kanchanaburi Province, Thailand (1.0 PP, 100 UFB; Fig. 2). The phylogenies also revealed that the current concept of *C.oldhami* is non-monophyletic.

The uncorrected pairwise sequence divergences (*p*-distances) between the Si Sawat species and all others in the *C.oldhami* species group used in this study are given in Table 3. The sequence divergences within the Si Sawat species were low, ranging from 0.0–1.2% (mean = 0.3%). However, the Si Sawat species had uncorrected *p*-distances of 7.7–17.7% from other members of the *C.oldhami* group, 7.7–8.0% (mean = 7.7%) from the sister taxon *Cyrtodactylus* sp. MT468911, and 17.3–17.7% (mean = 17.6%) from *C.zebraicus*, which it closely resembles in color pattern (Table 3).

**Table 3.** Uncorrected pairwise sequence divergences (*p*-distances) in the mitochondrial NADH dehydrogenase subunit 2 gene and flanking tRNA regions of *Cyrtodactylusmonilatus* sp. nov. and related species. 1 = *Cyrtodactylusmonilatus* sp. nov., 2 = *Cyrtodactylus* sp. MT468911, 3 = Cyrtodactyluscf.oldhami, 4 = *Cyrtodactylus* sp. MT 468910, 5 = *Cyrtodactylus* sp. MW713969, 6 = *C.thirakhupti*, 7 = *C.payarhtanensis*, 8 = *C.sanook*, 9 = *C.oldhami*MF872302, 10 = *C.oldhami*MF872301, 11 = *C.oldhami*MH940241, 12 = *C.saiyok*, 13 = *C.lenya*, and 14 = *C.zebraicus*.

No.	1	2	3	4	5	6	7	8	9	10	11	12	13	14
**1**	0.3													
(*N* = 9)	(0.0–1.2)													
**2**	7.7	–												
(*N* = 1)	(7.7–8.0)													
**3**	11.5	10.8	–											
(*N* = 1)	(11.2–11.7)	(10.8)												
**4**	11.6	11.2	1.8	–										
(*N* = 1)	(11.4–11.7)	(11.2)	(1.8)											
**5**	11.8	10.0	9.3	9.3	–									
(*N* = 1)	(11.7–12.0)	(10.0)	(9.3)	(9.3)										
**6**	12.0	11.3	9.0	9.2	9.7	0.0								
(*N* = 2)	(11.4–12.4)	(11.1–11.4)	(9.0)	(9.1–9.2)	(9.7)	(0.0)								
**7**	12.3	11.1	10.6	10.9	7.4	8.3	0.1							
(*N* = 3)	(11.7–13.2)	(10.7–11.9)	(10.2–11.4)	(10.5–11.6)	(7.1–8.1)	(7.7–9.7)	(0.0–0.1)							
**8**	12.7	12.1	9.9	9.6	6.0	7.1	8.02	–						
(*N* = 1)	(12.6–12.8)	(12.1)	(9.9)	(9.6)	(6.0)	(7.0–7.2)	(7.7–8.7)							
**9**	12.8	11.3	9.7	9.9	6.5	6.9	7.7	7.7	–					
(*N* = 1)	(12.7–12.9)	(11.3)	(9.7)	(9.9)	(6.5)	(6.6–7.2)	(7.4–8.3)	(7.7)						
**10**	12.3	11.6	9.7	9.53	5.49	7.17	9.1	7.4	7.8	–				
(*N* = 1)	(12.13–12.57)	(11.58)	(9.7)	(9.53)	(5.49)	(6.61–7.18)	(8.63–9.97)	(7.43)	(7.8)					
**11**	13.0	12.1	9.3	9.3	10.7	10.8	11.2	11.0	10.6	11.4	–			
(*N* = 1)	(12.7–13.2)	(12.1)	(9.3)	(9.3)	(10.7)	(10.4–11.2)	(10.9–11.7)	(11.0)	(10.6)	(11.4)				
**12**	15.4	14.6	14.2	14.8	14.4	14.9	14.8	15.1	14.6	15.5	15.3	0.1		
(*N* = 3)	(15.2–15.6)	(14.3 –14.8)	(14.1–14.3)	(14.7–14.9)	(14.2–14.6)	(14.6–15.2)	(14.1–16.4)	(14.9–15.3)	(14.4–14.8)	(15.2–15.8)	(15.0–15.5)	(0.1–0.2)		
**13**	16.	15.1	16.5	16.6	15.5	14.9	16.3	16.9	16.1	16.7	17.5	16.1	0.5	
(*N* = 3)	(16.6–17.0)	(15.0–15.1)	(16.4–16.6)	(16.4–16.7)	(15.4–15.7)	(14.1–15.6)	(15.6–17.6)	(16.7–17.1)	(16.0–16.1)	(16.6–16.8)	(17.3–17.6)	(15.7–16.4)	(0.2–0.6)	
**14**	17.6	16.3	16.8	17.0	16.9	16.3	17.7	17.5	17.3	17.2	17.4	17.2	13.2	6.0
(*N* = 2)	(17.3–17.7)	(16.2–16.3)	(16.6–16.9)	(16.9–17.0)	(16.8–16.9)	(15.3–17.2)	(16.7–19.2)	(17.5)	(17.2–17.4)	(17.2–17.4)	(17.2–17.6)	(15.7–16.4)	(13.0–13.2)	(6.0)

### ﻿Taxonomy

Based on the results of mtDNA and morphological comparisons (see below), the *Cyrtodactylus* specimens from Si Sawat District, Kanchanaburi Province, western Thailand distinctly differed from *C.zebraicus* and other species of the *oldhami* group. Thus, we hypothesize that the Si Sawat specimens represent a distinct species that is described as new, as follows.

#### ﻿ Cyrtodactylus monilatus sp. nov.

Taxon classificationAnimaliaSquamataGekkonidae

1A2D6396-16A0-53F4-B53C-B247A70819B4

http://zoobank.org/8F2DB395-0234-47D5-B272-0778D34ABE95

**Figure 3.**
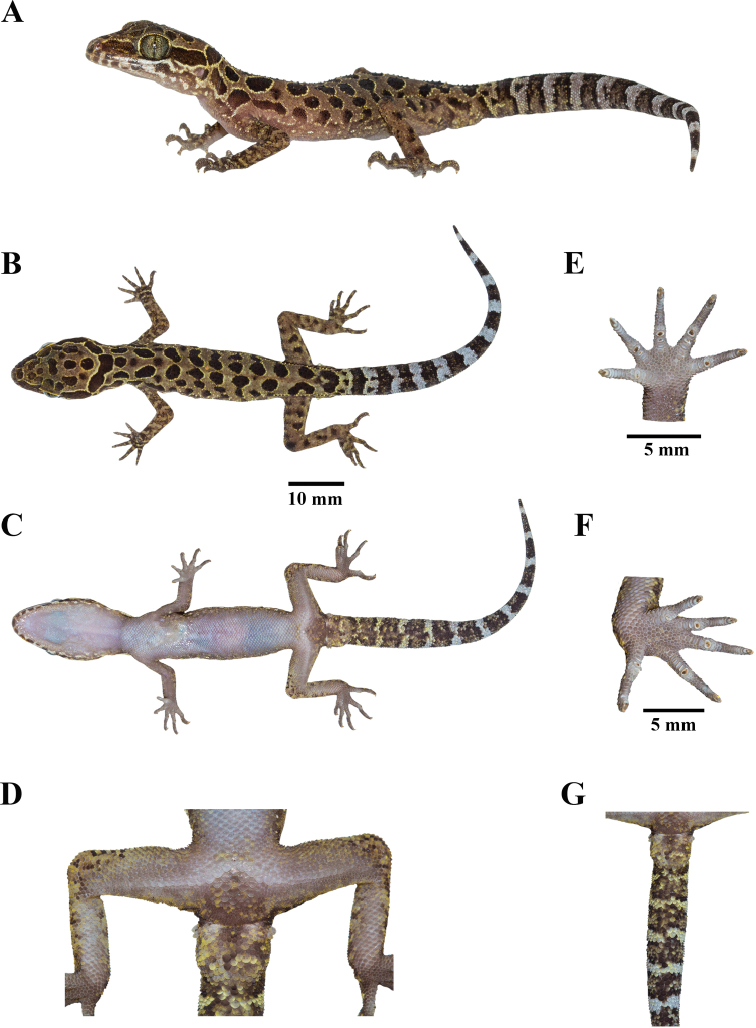
Adult male holotype of *Cyrtodactylusmonilatus* sp. nov. (ZMKU R 00943) in life from Tham Phrathat Protection Unit, Si Sawat District, Kanchanaburi Province, Thailand **A** lateral view **B** dorsal view **C** ventral view **D** precloacal region showing distribution of continuous, enlarged femoroprecloacal scales **E** palmar view of the left hand **F** plantar view of the left foot, and **G** ventral view of tail showing not enlarged median subcaudal scales.

**Figure 4.**
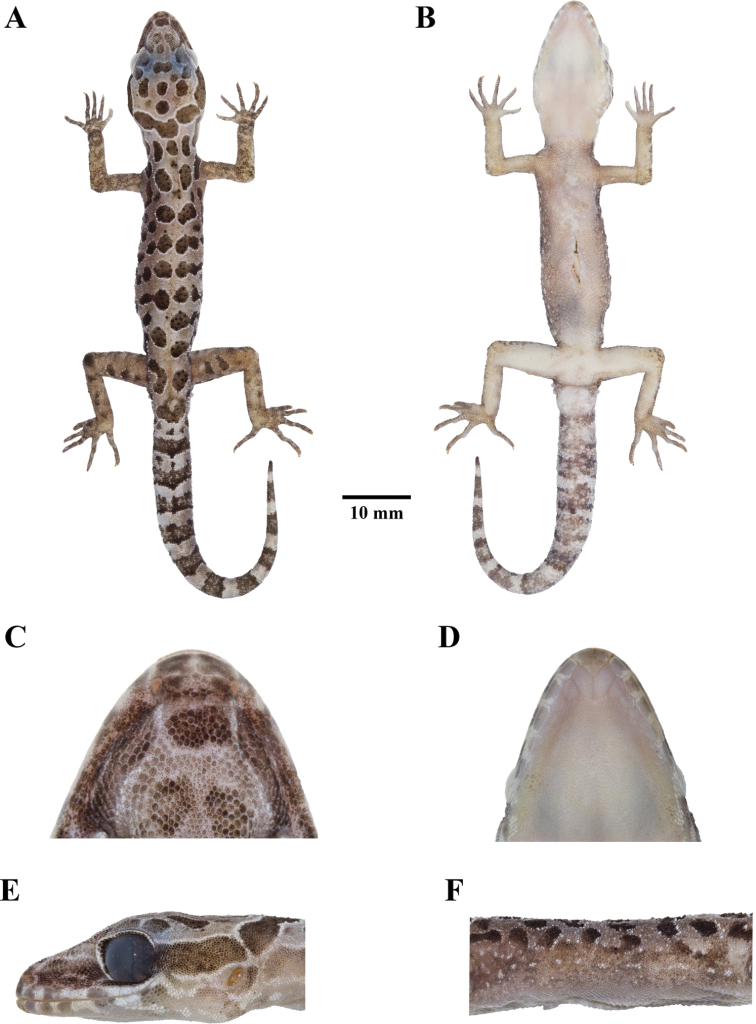
Adult male holotype of *Cyrtodactylusmonilatus* sp. nov. (ZMKU R 00943) in preservation **A** dorsal view **B** ventral view **C** dorsal view showing the rostral, supranasal, and internasal scales **D** ventral view showing the mental and postmental scales **E** lateral view of head of the left side **F** lateral view of flank of the left side.

**Figure 5.**
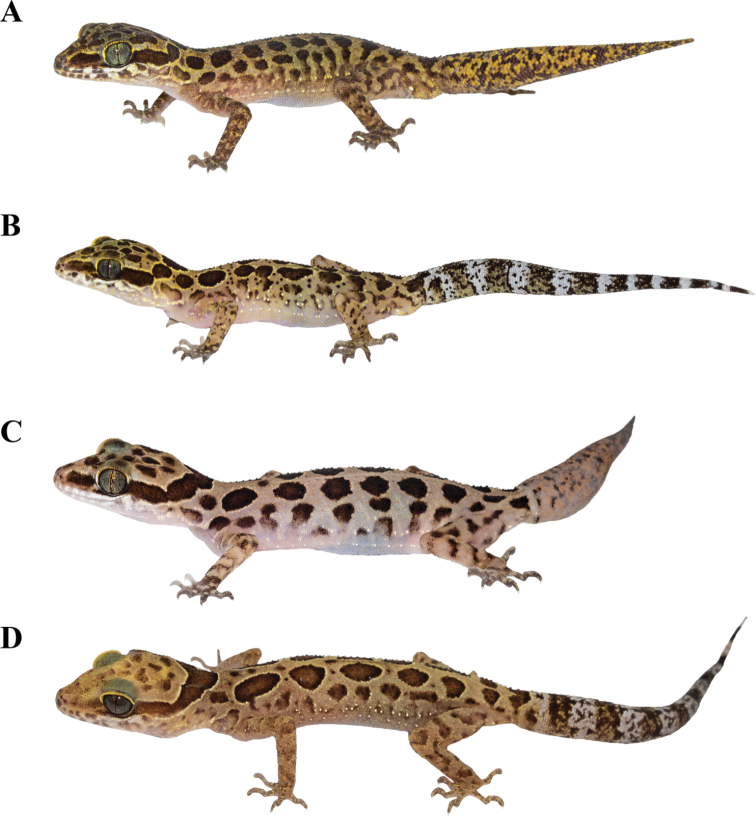
Paratypes of *Cyrtodactylusmonilatus* sp. nov. in life showing variation in color pattern **A** adult male (ZMKU R 00935) **B** adult male (ZMKU R 00944) from Tham Phrathat Protection Unit **C** adult female (ZMKU R 00927) from Erawan Waterfall **D** adult female (ZMKU R 00926) from Tham Than Lot Noi-Tham Than Lot Yai Nature Trail, Si Sawat District, Kanchanaburi Province, Thailand.

Common English name: Kanchanaburi Spotted Bent-toed Gecko Figs 3 , 4 , 5 , 7

##### Material examined.

***Holotype*.**ZMKU R 00943, adult male (Figs 3, 4), collected from Thailand, Kanchanaburi Province, Si Sawat District, Tha Kradan Subdistrict, Erawan National Park, Tham (= cave) Phrathat Protection Unit (14°23.754'N, 99°04.751'E, 699 m elevation), 19 November 2021, by Siriporn Yodthong, Attapol Rujirawan, Akrachai Aksornneam, and Natee Ampai.

***Paratypes*** (Fig. 5). Seven adult males (ZMKU R 00934–00939, ZMKU R 00944) and three adult females (ZMKU R 00940–00942), same data as holotype. Three adult males (ZMKU R 00928–00930) and two adult females (ZMKU R 00931–00932), same data as holotype except collected on 26 November 2019, by Siriporn Yodthong, Attapol Rujirawan, Akrachai Aksornneam, and Korkhwan Termprayoon. One adult female (ZMKU R 00927), collected from Thailand, Kanchanaburi Province, Si Sawat District, Tha Kradan Subdistrict, Erawan National Park, Erawan Waterfall (14°22.315'N, 99°08.806'E, 82 m elevation) on 25 November 2019 by Siriporn Yodthong, Attapol Rujirawan, Akrachai Aksornneam, and Korkhwan Termprayoon. Three adult females (ZMKU R 00924–00926), collected from Thailand, Kanchanaburi Province, Si Sawat District, Khao Chot Subdistrict, Chaloem Ratanakosin National Park, Tham Than Lot Noi-Tham Than Lot Yai Nature Trail (14°40.158'N, 99°17.436'E, 526 m elevation) on 20 April 2019 by Siriporn Yodthong, Akrachai Aksornneam, Korkhwan Termprayoon, and Natee Ampai.

##### Referred specimens.

One juvenile (ZMKU R 00923), collected from Thailand, Kanchanaburi Province, Si Sawat District, Khao Chot Subdistrict, Chaloem Ratanakosin National Park, Tham Than Lot Noi-Tham Than Lot Yai Nature Trail (14° 39.767'N, 99°18.314'E, 233 m elevation) on 19 April 2019 by Siriporn Yodthong, Akrachai Aksornneam, Korkhwan Termprayoon, and Natee Ampai. One juvenile (ZMKU R 00933), same data as holotype except collected on 26 November 2019 by Siriporn Yodthong, Attapol Rujirawan, Akrachai Aksornneam, and Korkhwan Termprayoon.

##### Etymology.

The specific epithet *monilatus* is taken from *monile* (L.) for necklace or string of beads and *latus* (L.) for flank, in reference to the new species having two rows of small, diffuse, yellow or yellowish white spots on the flanks that resemble a beaded necklace. These spots are an important color pattern difference between the new species and *C.zebraicus*. We propose “Kanchanaburi Spotted Bent-toed Gecko” for the common English name and “ตุ๊กแกป่่่าลายจุดเมืองกาญจน์” (Took kae pa lai jud mueang kan) for the common Thai name of the new species.

##### Diagnosis.

*Cyrtodactylusmonilatus* sp. nov. is assigned to the *C.oldhami* group on the basis of its recovered phylogenetic position (Fig. 1). This species can be distinguished from all other species of the *C.oldhami* group (sensu Grismer et al. 2021b) by having the following combination of characters: (1) a medium-sized *Cyrtodactylus*, SVL 53.7–63.3 mm in adult males, 58.6–75.8 mm in adult females; (2) 10–13 supralabial and 8–11 infralabial scales; (3) 22–34 paravertebral tubercles; (4) 16–21 longitudinal rows of dorsal tubercles; (5) 34–42 ventral scales; (6) 12–16 total subdigital lamellae on the fourth finger; (7) 15–19 total subdigital lamellae on the fourth toe; (8) 30–39 contiguous enlarged femoroprecloacal scales; (9) femoral pores and precloacal pores absent in both sexes; (10) four or five rows of postprecloacal scales; (11) precloacal groove or depression absent; (12) enlarged median subcaudal scales absent; (13) 9–12 dark and light caudal bands encircling the original tail; (14) weak ventrolateral folds present; (15) subconical to slightly prominent trihedral keeled tubercles on body that extend past the base of the tail but no further than 1/3 of anterior portion of tail; (16) top of head bearing large, dark-brown blotches edged in yellow or yellowish white with no light-colored network; (17) 4–7 dorsal body bands composed of paired, paravertebral, dark-brown blotches edged in yellow or yellowish white; and (18) two rows of small, diffuse, yellow or yellowish white spots on flanks.

##### Description of holotype

(Figs 3, 4). Adult male with 56.4 mm SVL; head moderate in length (HL/SVL 0.29), wide (HW/HL 0.65), slightly flattened (HD/HL 0.40), distinct from neck, and triangular in dorsal profile; lores concave anteriorly, inflated posteriorly; frontal region flattened, prefrontal region slightly concave, canthus rostralis rounded; snout rather elongate (ES/HL 0.40), rounded in rostral region, eye to snout distance slightly greater than head depth; eye large (ED/HL 0.29), eyeball slightly protuberant, eye diameter less than the eye to ear distance, pupil vertical; ear opening elliptical, obliquely oriented, moderate in size (EL/HL 0.09); eye to ear distance greater than eye diameter; rostral large, subrectangular, height 1.6 mm, shorter than wide, 2.8 mm, medially divided by dorsal a furrow, reaching to approximately half-way down rostral height, bordered posteriorly by supranasals and internasal, laterally by first supralabials and nostrils; external nares at anterior angle of snout, directed lateroposteriorly, bordered anteriorly by rostral, dorsally by large supranasal, posteriorly by two small postnasals, ventrally by first supralabial; internarial distance narrow; supranasals subrectangular, separated by two small internasals, bordered anteriorly by rostral, laterally by nostrils, posteriorly by four small scales; two internasals, subpentagonal, vertically arranged, slightly protruding rostral, bordered posteriorly by three small scales; 8/7 (right/left) supralabials extending to below midpoint of eye, 12/11 to below the posterior margin of the eyeball, subrectangular anteriorly, elliptical shape posteriorly; 5/7 infralabials extending to below midpoint of eye, 9/10 to below the posterior margin of the eyeball, larger than supralabials, tapering smoothly posteriorly; scales of frontonasal, prefrontal and lores, small, relatively raised, domed, slightly larger than granular scales on top of head and occiput; scales on occiput intermixed with scattered, slightly larger, more rounded, dome to subconical tubercles, more prominent tubercles between occiput and above ear opening; dorsal supraciliaries crenulated, not elongate or keeled; mental large, triangular, 2.4 mm in width, 1.8 mm in length, bordered laterally by first infralabials and posteriorly by large, right and left trapezoidal postmentals that contact medially for 66% of their length posterior to mental; one row of slightly enlarged chin shields extending posteriorly to sixth (right) and seventh (left) infralabial; and gular and throat scales small, granular, grading posteriorly into larger, flat, smooth, imbricate, pectoral and ventral scales.

Body slender, relatively short (AG/SVL 0.44), with weak ventrolateral folds; scales on dorsum small, mostly homogenous, granular, interspersed with larger, irregularly arranged, slightly prominent trihedral keeled tubercles; tubercles extending from occiput beyond to the base of the tail but not farther than 1/3 of tail; tubercles on occiput, nape and anterior of body at level above shoulder smaller, subconical; those mid-dorsally and on the posterior section of the body larger, being more dense, slightly more prominently keeled, and more regularly arrange in sacral region and tail base; tubercles on flanks sparse; approximately 16 longitudinal rows of dorsal tubercles; approximately 28 paravertebral tubercles; 38 flat, imbricate, smooth ventral scales, those near midline larger than those laterally and dorsal scales; femoral scales enlarged, extending along 2/3 of femora and contiguous with enlarged precloacal scales; precloacal scales smooth, approximately twice the size of femoral scales; 33 contiguous femoroprecloacal scales; femoral pores and precloacal pores absent; four rows of enlarged postprecloacal scales; and precloacal groove or depression absent.

Limbs moderately slender; forelimbs relatively short (FL/SVL 0.15); scales on dorsal surface domed to subconical, granular, slightly larger than those on body, interspersed with sparsely enlarged, subconical and trihedrally keeled tubercles; dorsal scales of wrist and palm flat, smooth, round, imbricate; ventral scales of palm flat, weakly rounded, slightly raised, not imbricate, smaller than those on body; 16/16 (right/left) total subdigital lamellae on fourth finger, 4/4 proximal subdigital lamellae rectangular with rounded to weakly rounded corners, broadly expanded proximal to joint inflection on fourth finger, 12/12 distal subdigital lamellae, slightly expanded immediately distal to joint, becoming gradually more expanded near the claw; digits well-developed, relatively long, inflected at basal interphalangeal joints; digits slightly narrower distal to inflections; no interdigital webbing; claw well-developed, relatively short, claw base sheathed by a dorsal and ventral scales; hind limbs more robust than forelimbs, moderate in length (TBL/SVL 0.18); dorsal scales domed to subconical, granular, interspersed with enlarged subconical and trihedrally keeled tubercles, and anterior part of thigh covered by flat, slightly larger, imbricate scales; ventral scales of femora flat, smooth, imbricate, smaller than those on body; small postfemoral scales form an abrupt union with large, flat ventral scales of posteroventral margin of thigh; ventral scales of tibia flat, imbricate; dorsal scales of plantar surface relatively smooth, rounded, imbricate; ventral scales of plantar surface low flat, weakly rounded; 18/19 (right/left) total subdigital lamellae on fourth toe, 5/6 proximal subdigital lamellae, rectangular with rounded to weaky rounded corners, broadly expanded proximal to joint inflection on fourth toe, 13/13 distal subdigital lamellae, slightly expanded immediately distal to joint, becoming gradually more expanded near the claw; digits well-developed, relatively long, inflected at basal, interphalangeal joints; and claw well-developed, relatively short, claw base sheathed by a dorsal and ventral scales.

Tail 58.1 mm in length, original, slightly longer than SVL (TL/SVL 1.03), moderate in proportions, segmented, cylindrical, wide anteriorly, 4.6 mm in width at base, tapering to a tip, covered with small scales on the dorsal surface but slightly larger scales on ventral surface; dorsal scales of tail base granular, round, becoming larger, flatter, subimbricate posteriorly; those on tail base bearing trihedrally keeled tubercles forming paravertebral rows, four dorsal longitudinal tubercles rows, two transverse rows of dorsal tubercles extend from tail base to posterior margin of third caudal band, 7.1 mm from tail base, approximately 1/8 of tail; no enlarged median row of transverse scales on subcaudal region; no caudal furrow; base of tail forming hemipenial swelling; 2/2 (right/left) postcloacal tubercles on the enlarged smooth hemipenial swelling; and postcloacal tubercles approximately equal size.

##### Coloration of holotype in life

**(Fig. 3).** Dorsal ground color of head, body, and limbs yellowish brown; top of head bearing large, dark-brown blotches edged in yellow; superciliary scales yellow; wide dark-brown stripe edged in yellow on canthus, extending from posterior margin of nostril to anterior margin of orbit; wide, discontinuous, dark-brown nuchal loop edged in yellow, extending from posterior margin of one orbit, across occiput to posterior margin of the other orbit; three large, dark-brown blotches edged in yellow on nape; seven paravertebral blotches on right and six on left between limb insertions resulting in five anterior bands of paired, paravertebral, dark-brown blotches, remaining bands composed of one and two, unpaired, paravertebral, blotches; all dorsal bands terminate on upper flanks with a series of dark-brown, irregularly shaped blotches of varying sizes edged in yellow and yellowish white; two similarly colored postsacral bands, anterior composed of paravertebral blotches and posterior composed of confluent blotches; 11 dark-brown caudal bands and 11 white caudal bands; all caudal bands encircle the tail; dorsal portion of forelimbs bearing irregularly shaped dark markings with dull-yellow spots; dorsal portion of hind limbs mottled with yellow spots and small, poorly defined, dark-brown blotches; supralabial and infralabial scales off-white with darker markings; suborbital region to forelimb insertions covered with irregularly shaped dark-brown and yellowish white markings; lower flanks bearing two rows of small, diffuse, yellowish white spots; all ventral surfaces generally greyish white, immaculate, except for ventral surface of knee, precloacal and postcloacal regions, and hemipenial swellings which bear dark and yellow to yellowish white markings.

##### Coloration of holotype in preservation

**(Fig. 4).** Color pattern of head, body, limbs, and tail similar to that in life with some fading. Ground color of head, body, and limbs light-beige; dark body and dark caudal bands lighter than in life; yellow coloration on dorsal and ventral surface fade to off-white; and all ventral surfaces light-beige.

##### Variations.

Morphometric, meristic and color pattern characters of the type series and referred specimens of *C.monilatus* sp. nov. are presented in Tables 4–6. All paratypes approximate the holotype in general aspects of morphology, with variations in coloration and banding (Fig. 5). Dorsal ground color varies from beige, brown, to yellowish brown. Edge of dark-brown blotches on dorsum varies from yellow to yellowish white. Pattern on top of head of one specimen (ZMKU R 00926) has faint, poorly-defined, dark, irregularly shaped blotches. One specimen (ZMKU R 00926) has a faint, poorly-defined, dark stripe on canthus region. Nuchal loop patterns of three specimens (ZMKU R 00925–00927) are completely continuous. Dorsal body bands of one specimen (ZMKU R 00925) comprise four bands, seven specimens (ZMKU R 00923, ZMKU R 00924, ZMKU R 00926, ZMKU R 00929, ZMKU R 00933, ZMKU R 00941 and ZMKU R 00944) have five bands, and nine specimens (ZMKU R00927, ZMKU R 00930–00931, ZMKU R 00934, ZMKU R 00937–00940 and ZMKU R 00942) have six bands. Ventral ground color varies from beige to greyish white.

**Table 4.** Descriptive measurements in millimeters of the type series of *Cyrtodactylusmonilatus* sp. nov. Abbreviations are defined in the text.

Characters	Holotype male	Holotype and paratype males		Paratype females	
	*N* = 1	*N* = 11		*N* = 9	
		Min–Max	Mean ± SD	Min–Max	Mean ± SD
**SVL**	56.4	53.7–63.3	58.0 ± 3.4	58.6–75.8	68.7 ± 5.6
**HL**	16.4	15.5–18.10	16.6 ± 0.9	16.8–22.0	19.3 ± 1.7
**HW**	10.6	10.1–12.1	11.0 ± 0.6	11.5–15.4	13.3 ± 1.2
**HD**	6.5	5.8–7.5	6.6 ± 0.6	6.4–9.1	7.7 ± 0.9
**ED**	4.8	4.3–5.3	4.7 ± 0.4	4.8–5.4	5.2 ± 0.3
**EE**	5.1	4.0–5.3	4.9 ± 0.4	5.1–6.7	5.9 ± 0.6
**ES**	6.6	5.9–7.4	6.6 ± 0.4	6.5–8.8	7.4 ± 0.7
**EN**	5.2	4.3–5.4	4.9 ± 0.3	4.8–6.5	5.5 ± 0.5
**IO**	5.3	5.1–6.4	5.7 ± 0.4	5.7–7.6	6.6 ± 0.7
**EL**	1.5	1.1–1.8	1.4 ± 0.2	1.3–1.7	1.5 ± 0.2
**IN**	1.9	1.7–2.1	1.9 ± 0.1	1.8–2.4	2.1 ± 0.2
**AG**	24.7	22.3–27.8	25.4 ± 1.7	26.1–34.4	31.1 ± 2.8
**FL**	8.3	8.0–9.6	8.8 ± 0.5	9.1–11.8	10.4 ± 1.0
**TBL**	10.4	9.8–11.7	10.8 ± 0.7	11.4–14.1	12.8 ± 1.0
**TL (original)**	58.1	58.1–62.10^a^	59.9 ± 1.7^a^	64.0–77.7^c^	71.4 ± 5.7^c^
**TL (regenerated)**	NA	41.8–60.0^b^	49.5 ± 5.8^b^	25.7–55.1^d^	42.8 ± 12.9^d^
**TW**	4.6	4.0–5.4	4.9 ± 0.5	4.5–5.3	4.9 ± 0.3
**TD**	4.0	4.0–5.6	4.7 ± 0.5	4.3–5.4	4.8 ± 0.4

^a^*N* = 4; ^b^*N* = 7; ^c^*N* = 5; ^d^*N* = 4

**Table 5.** Meristic characters (right/left) and color patterns of *Cyrtodactylusmonilatus* sp. nov. Abbreviations are defined in the text. Key: NA = data unavailable or unapplicable.

Characters	Holotype	Holotype and paratypes
	*N* = 1	*N* = 20
		Min–Max
SL	12/11	10–13
SL-mideye	8/7	6–9
IL	9/10	8–11
IL-mideye	5/7	5–8
Body tubercles pointed and keeled	Yes	Yes
PVT	28	25–34
LRT	16	16–21
VS	38	34–42
4FLU	12/12	9–12
4FLE	4/4	3–5
4FL	16/16	12–16
4TLU	13/13	10–13
4TLE	5/6	4–6
4TL	18/19	15–19
Enlarge femoral and precloacal scales continuous	Yes	Yes
FPS	33	30–39*
PP	Absent	Absent
PPS	4	4–5
PPT	2/2	2–3
Enlarged median subcaudal scales	No	No
Nuchal loop discontinuous	Yes	Yes & No
Paravertebral elements not in contact	Yes	Yes
BB	6	4–7
DCB	11	9–12
LCB	11	9–12

* *N* = 19, data from ZMKU R 00934 was not included because its FPS has a defect.

**Table 6.** Meristic characters (right/left) and color patterns of the referred specimens of *Cyrtodactylusmonilatus* sp. nov. Abbreviations are defined in the text. Key: NA = data unavailable or unapplicable.

Characters	ZMKU R 00933	ZMKU R 00923	Min–Max
Age	Juvenile	Juvenile	*N* = 2
SVL	40.6	31.3	31.3–40.6
SL	12/11	12/10	10–12
SL-mideye	8/7	8/7	7–8
IL	10/9	8/8	8–10
IL-mideye	7/6	5/6	5–7
Body tubercles pointed and keeled	Yes	Yes	Yes
PVT	22	27	22–27
LRT	16	17	16–17
VS	34	40	34–40
4FLU	11/11	10/10	10–11
4FLE	4/4	4/4	4
4FL	15/15	14/14	14–15
4TLU	12/12	11/11	11–12
4TLE	6/5	6/6	5–6
4TL	18/17	17/17	17–18
Enlarge femoral and precloacal scales continuous	Yes	Yes	Yes
FPS	33	33	33
PPS	5	5	5
PPT	2/3	2/2	2–3
Enlarged median subcaudal scales	NA	No	No
Nuchal loop discontinuous	Yes	Yes	Yes
Paravertebral elements not in contact	Yes	Yes	Yes
BB	6	5	5–6
DCB	NA	10	10
LCB	NA	11	11

Internasal scales of ten specimens (ZMKU R 00923–00924, ZMKU R 00926, ZMKU R 00930, ZMKU R 00933, ZMKU R 00935, ZMKU R 00937, ZMKU R 00940–00941, ZMKU R 00944) are single and eight specimens (ZMKU R 00925, ZMKU R 00928–00929, ZMKU R 00932, ZMKU R 00934, ZMKU R 00936, ZMKU R 00938, ZMKU R 00942) are absent. Regenerated tail covered with flat, imbricate, round scales; enlarge median subcaudal scales absent; ground color of regenerated tail varies from beige, yellowish brown, brown to dark-brown bearing brown and dark markings; dark and light caudal bands absent (Fig. 5). Females have larger body SVL than males (Table 4). In life, coloration and banding pattern of juvenile specimens (ZMKU R 00923 and ZMKU R 00933) resemble that of the adults.

##### Distribution and natural history.

**Figure 6.**
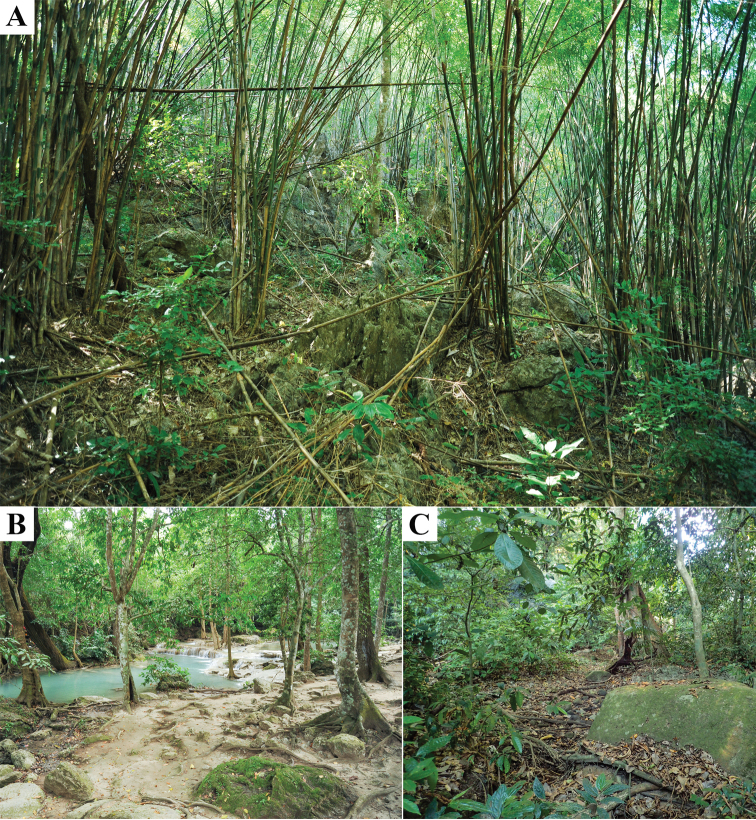
Sampling localities of *Cyrtodactylusmonilatus* sp. nov. **A** the type locality in Tham Phrathat Protection Unit **B** Erawan Waterfall **C** Tham Than Lot Noi-Tham Than Lot Yai Nature Trail, Si Sawat District, Kanchanaburi Province, Thailand.

*Cyrtodactylusmonilatus* sp. nov. is currently known from only three localities in Si Sawat District, Kanchanaburi Province, western Thailand: Tham Phrathat Protection Unit, Erawan Waterfall in Erawan National Park, and Tham Than Lot Noi-Tham Than Lot Yai Nature Trail in Chaloem Rattanakosin National Park (Figs 1, 6). All individuals were found in karst forests with mixed deciduous trees, and dry evergreen trees at 82–699 m elevation. These areas are surrounded by agricultural lands (orchards, rubber plantation, and pasture lands) and human residential areas. Specimens (*N* = 22) were collected at night (1900–2100 hr) during the dry season (November–April) on the forest floor (54.6%; *N* = 12), on karst boulder outcrops (22.7%; *N* = 5), and on shrub or bamboo twigs with ≤ 100 cm above ground level (22.7%; *N* = 5). The range in altitude at which the specimens were collected suggests that elevation has little to do with their distribution. It is likely that karst forests are the ecological factor that determines where they occur.

At Tham Phrathat Protection Unit, the holotype (ZMKU R 00943) was found on 19 November 2021 on the forest floor covered with leaf litter, at a temperature 24.0 °C and relative humidity 90.0%. On the previous day with temperatures between 24.2–24.4 °C and relative humidity between 82.6–83.9%, three specimens (ZMKU R 00937, ZMKU R 00939–00940) were found on the forest floor covered with leaf litter, two specimens (ZMKU R 00934–00935) were found on shrub twigs with ≤ 10 cm above ground level, and three specimens (ZMKU R 00936, ZMKU R 00938 and ZMKU R 00941) were found on the karst boulder outcrops, including one gravid female (ZMKU R 00942) containing two eggs (externally visible). Juveniles and immatures (SVL < 50 mm) were found on the forest floor and on the karst boulder outcrops but not collected. During November of the previous year (2019) at a temperature 25.9 °C and relative humidity of 54.3%, one specimen (ZMKU R 00929) was found on the twig of a shrub approximately 30 cm above ground level, another specimen (ZMKU R 00930) was found on bamboo twig around 100 cm above ground level, and three specimens (ZMKU R 00928, ZMKU R 00931–00932) were found on the forest floor covered with leaf litter, including one juvenile (ZMKU R 00933). Other sympatric lizard species found at this locality included *Acanthosauracrucigera* Boulenger, 1885, *Cnemaspishuaseesom* Grismer, Sumontha, Cota, Grismer, Wood, Pauwels & Kunya, 2010, *Cyrtodactylustigroides* Bauer, Sumontha & Pauwels, 2003, *Dixoniushangseesom* Bauer, Sumontha, Grossmann, Pauwels & Vogel, 2004, *Dixoniussiamensis* (Boulenger, 1899), *Eutropismacularia* (Blyth, 1853), *Gehyramutilata* (Wiegmann, 1834), and *Subdolusepsbowringii* (Günther, 1864).

**Figure 7.**
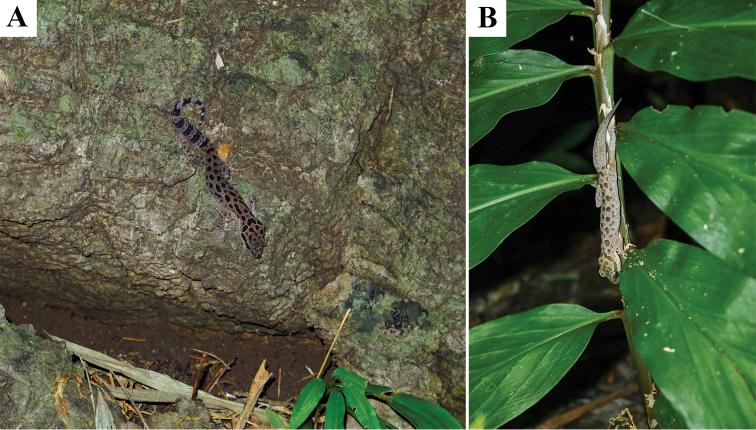
Habitat of *Cyrtodactylusmonilatus* sp. nov. Tham Phrathat Protection Unit, Si Sawat District, Kanchanaburi Province, Thailand **A** adult female (ZMKU R 00941) on boulder outcrops **B** adult male (not collected) on shrub.

At Erawan Waterfall, one gravid female (ZMKU R 00927) contained two eggs (externally visible) and was found on the forest floor near the waterfall stream during November 2019. Other sympatric lizard species found at this locality included *Dracotaeniopterus* (Günther, 1861) and *Sphenomorphusmaculatus* (Blyth, 1853).

At Tham Than Lot Noi-Tham Than Lot Yai Nature Trail, one juvenile specimen (ZMKU R 00923) was found on karst boulder outcrops at a temperature 27.1 °C and relative humidity 72.0%, another adult female (ZMKU R 00925) was found on dry twig on the forest floor, and one gravid female (ZMKU R 00926) containing two eggs (externally visible) was found on the forest floor covered with leaf letter at a temperature 31.9 °C and relative humidity 56.9%. Other sympatric lizard species found at this locality included *Cyrtodactylus* sp., *Dixoniussiamensis*, *Dracotaeniopterus*, and *Sphenomorphusmaculatus*.

##### Comparisons.

*Cyrtodactylusmonilatus* sp. nov. is differentiated from all seven species of *C.oldhami* group and two additional species, *C.phetchaburiensis* and *C.surin* by having a unique combination of morphological characters, its phylogenetic placement (Fig. 2), and having uncorrected pairwise sequence divergences in mtDNA from all other members of the *oldhami* group of 7.7–17.7%, (Table 3).

*Cyrtodactylusmonilatus* sp. nov. differs from *C.lenya* Mulcahy, Thura & Zug, 2017 by having 25–34 paravertebral tubercles (vs. 39–41 tubercles); 34–42 ventral scales (vs. 29 scales); enlarged median subcaudal scales absent (vs. present); top of head bearing dark-brown blotches edged in yellow or yellowish white (vs. indistinctly mottled); dorsal body bands composed of paired, paravertebral, dark-brown blotches edged in yellow or yellowish white (vs. broad dark-brown dorsal bands with narrow chocolate brown borders fore and aft, alternating with narrower medium to light-brown interspaces); and two rows of small, diffuse, yellow or yellowish white spots on flanks present (vs. absent).

*Cyrtodactylusmonilatus* sp. nov. differs from *C.oldhami* (Theobald, 1876) by having 16–21 longitudinal rows of dorsal tubercles (vs. 30 rows); precloacal pores absent in both sexes (vs. present in males); top of head bearing large, dark-brown blotches edged in yellow or yellowish white (vs. uniform brown); and dorsal body bands composed of paired, paravertebral, dark-brown blotches edged in yellow or yellowish white (vs. elongated or rounded spots arranged in four longitudinal lines).

*Cyrtodactylusmonilatus* sp. nov. differs from *C.payarhtanensis* Mulcahy, Thura & Zug, 2017 by being smaller, SVL 53.7–63.3 mm in adult males, 58.6–75.8 mm in adult females (vs. 61–80 mm in adult males, 74–83 mm in adult females); 22–34 paravertebral tubercles (vs. 40–45 tubercles); 34–42 ventral scales (vs. 26–32 scales); 15–19 total subdigital lamellae on the fourth toe (vs. 12 or 13); enlarged median subcaudal scales absent (vs. present); top of head bearing dark-brown blotches edged in yellow or yellowish white (vs. indistinctly mottled, dusky brown marks); dorsal body bands composed of paired, paravertebral, dark-brown blotches edged in yellow or yellowish white (vs. irregularly shaped and edged dark-brown); and two rows of small, diffuse, yellow or yellowish white spots on flanks present (vs. absent).

*Cyrtodactylusmonilatus* sp. nov. differs from *C.phetchaburiensis* Pauwels, Sumontha & Bauer, 2016 which is not in the phylogeny by lacking precloacal pores in both sexes (vs. present in males); enlarged median subcaudal scales absent (vs. present); and dorsal body bands composed of paired, paravertebral, dark-brown blotches edged in yellow or yellowish white (vs. absent).

*Cyrtodactylusmonilatus* sp. nov. differs from *C.saiyok* Panitvong, Sumontha, Tunprasert & Pauwels, 2014 by having 34–42 ventral scales (vs. 23–24 scales); precloacal pores absent in both sexes (vs. present in males); enlarged median subcaudal scales absent (vs. present); and dorsal body bands composed of paired, paravertebral, dark-brown blotches edged in yellow or yellowish white (vs. irregular, medially interrupted or not, black).

*Cyrtodactylusmonilatus* sp. nov. differs from *C.sanook* Pauwels, Sumontha, Latinne & Grismer, 2013 by being smaller, SVL 53.7–63.3 mm in adult males (vs. 72.9–79.5 mm); precloacal pores absent in both sexes (vs. present in males); enlarged median subcaudal scales absent (vs. present); and dorsal body bands composed of paired, paravertebral, dark-brown blotches edged in thin yellow or yellowish white (vs. irregular pale narrow bands).

*Cyrtodactylusmonilatus* sp. nov. differs from *C.surin* Chan-ard & Makchai, 2011 which is not in the phylogeny by having 34–42 ventral scales (vs. 25 ventral scales); enlarged median subcaudal scales absent (vs. present); and precloacal pores absent in both sexes (vs. present in males).

*Cyrtodactylusmonilatus* sp. nov. differs from *C.thirakhupti* Pauwels, Bauer, Sumontha & Chanhome, 2004 by being smaller, SVL 53.7–63.3 mm in adult males (vs. 72.0–79.6 mm in adult males); 16–21 longitudinal rows of dorsal tubercles (vs. 14 rows); enlarged median subcaudal scales absent (vs. present); and dorsal body bands composed of paired, paravertebral, dark-brown blotches edged in yellow or yellowish white (vs. yellowish bands with very dark brown borders).

**Figure 8.**
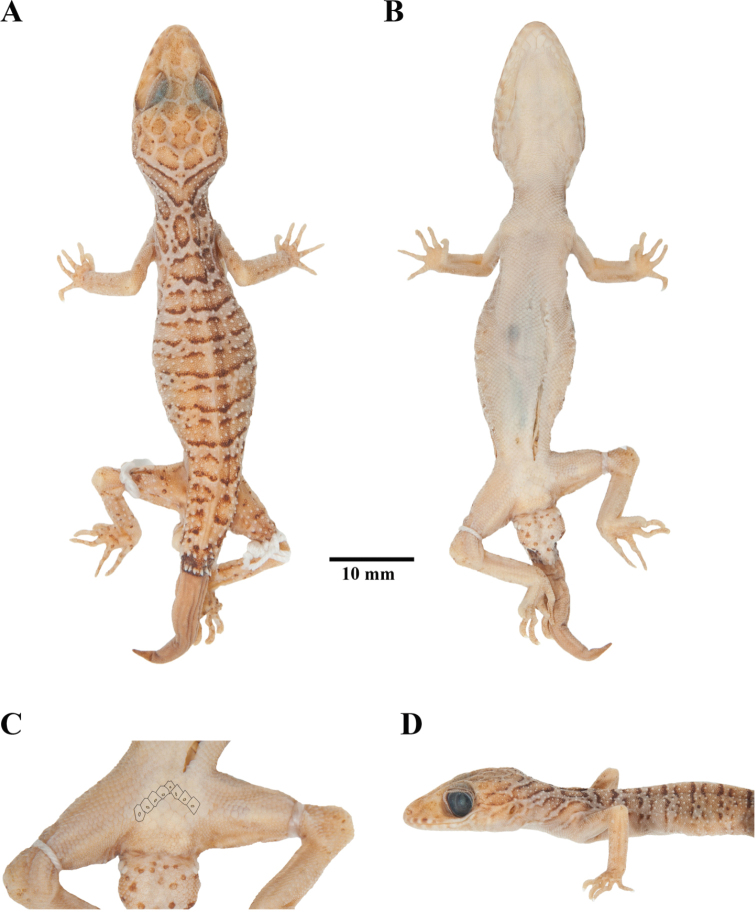
Adult male holotype of *Cyrtodactyluszebraicus* (FMNH 178286) from Ron Phibun District, Nakhon Si Thammarat Province, Thailand, in preservation **A** dorsal view **B** ventral view **C** precloacal region showing distribution of precloacal pores **D** lateral view of the left side.

*Cyrtodactylusmonilatus* sp. nov. differs from *C.zebraicus* Taylor, 1962 by having precloacal pores absent in both sexes (vs. present in males; Fig. 8); four or five rows of postprecloacal scales (vs. two rows); and two rows of small, diffuse, yellow or yellowish white spots on flanks present (vs. their absence; Fig. 8; Grismer et al. 2018a: fig. 4A; Bringsøe, 2020: fig. 1; Grismer et al. 2021b: fig. 28B).

## ﻿Discussion

The combination of morphological and molecular phylogenetic evidence in this study corroborated the hypothesis that the Si Sawat population should be recognized as a distinct species, described here as *Cyrtodactylusmonilatus* sp. nov., and that this new species is a member of the *C.oldhami* group. Morphologically, the new species superficially resembles *C.zebraicus* from southern Thailand in body shape and color pattern, but phylogenetically they are not closely related. Moreover, our phylogenetic analyses of the *C.oldhami* group indicated that populations in Thailand that are currently referred to *C.oldhami* are not monophyletic and likely represent additional, undescribed species (Chomdej et al. 2021; Grismer et al. 2021b). Unfortunately, the type locality of *C.oldhami* is uncertain (Annandale 1905, 1913; Das et al. 1998; Pauwels et al. 2016; Uetz et al. 2022) and so the concept of *C.oldhami* is limited to the original description of the holotype (Theobald 1876). It is therefore possible that none of the “*C.oldhami*” samples in our phylogenetic analyses represent true *C.oldhami*. Comparisons of the holotype to all specimens from Thailand and Myanmar currently referred to *C.oldhami* are necessary before further taxonomic partitioning of *C.oldhami* can be done.

*Cyrtodactylusphetchaburiensis* and *C.surin* were described from the Isthmus of Kra area (Phetchaburi Province and Surin Island, Phang-nga Provinces, respectively) based only on morphological data (Chan-ard and Makchai 2011; Pauwels et al. 2016). These two species can be tentatively assigned to the *C.oldhami* group based on their morphological appearances and geographic distributions (Pauwels et al. 2016; personal observation). However, genetic data are needed to verify their taxonomic status and phylogenetic placement. The discovery of *Cyrtodactylusmonilatus* sp. nov. brings the number of species in the *C.oldhami* group to eight and the total number of *Cyrtodactylus* in Thailand to 40 (Termprayoon et al. 2021; Uetz et al. 2022). *Cyrtodactylusmonilatus* sp. nov. is currently known only from the kart forests in Si Sawat District, Kanchanaburi Province in western Thailand. Additional field surveys and sampling in western Thailand and nearby areas including the Thai-Myanmar border, as well as re-evaluation of existing museum specimens, are needed to determine the actual geographic range of the new species.

## Supplementary Material

XML Treatment for Cyrtodactylus monilatus
